# Probiotic *Bacillus amyloliquefaciens* SC06 Induces Autophagy to Protect against Pathogens in Macrophages

**DOI:** 10.3389/fmicb.2017.00469

**Published:** 2017-03-22

**Authors:** Yanping Wu, Yang Wang, Hai Zou, Baikui Wang, Qiming Sun, Aikun Fu, Yuanyuan Wang, Yibing Wang, Xiaogang Xu, Weifen Li

**Affiliations:** ^1^Key Laboratory of Molecular Animal Nutrition of Ministry of Education, Institute of Feed Science, College of Animal Sciences, Zhejiang UniversityHangzhou, China; ^2^Department of Cardiology, Zhejiang Provincial People’s HospitalHangzhou, China; ^3^Department of Biochemistry, School of Medicine, Zhejiang UniversityHangzhou, China

**Keywords:** probiotics, *Bacillus amyloliquefaciens*, autophagy, pathogens, antibacterial activity, signaling pathways

## Abstract

Probiotics are increasingly applied in popularity in both humans and animals. Decades of research has revealed their beneficial effects, including the immune modulation in intestinal pathogens inhibition. Autophagy—a cellular process that involves the delivery of cytoplasmic proteins and organelles to the lysosome for degradation and recirculation—is essential to protect cells against bacterial pathogens. However, the mechanism of probiotics-mediated autophagy and its role in the elimination of pathogens are still unknown. Here, we evaluated *Bacillus amyloliquefaciens* SC06 (Ba)-induced autophagy and its antibacterial activity against *Escherichia coli* (*E. coli*) in murine macrophage cell line RAW264.7 cells. Western blotting and confocal laser scanning analysis showed that Ba activated autophagy in a dose- and time-dependent manner. Ba-induced autophagy was found to play a role in the elimination of intracellular bacteria when RAW264.7 cells were challenged with *E. coli*. Ba induced autophagy by increasing the expression of Beclin1 and *Atg5-Atg12-Atg16* complex, but not the AKT/mTOR signaling pathway. Moreover, Ba pretreatment attenuated the activation of JNK in RAW264.7 cells during *E. coli* infection, further indicating a protective role for probiotics via modulating macrophage immunity. The above findings highlight a novel mechanism underlying the antibacterial activity of probiotics. This study enriches the current knowledge on probiotics-mediated autophagy, and provides a new perspective on the prevention of bacterial infection in intestine, which further the application of probiotics in food products.

## Introduction

The gut is the largest immune organ in the body, where harbors a diverse array of organisms and the environment is quite complex (Ouwehand et al., 2005). Various factors such as foodborne pathogens may disturb the intestinal balance, leading to infectious diseases and diarrhea, which eventually influence the overall health (Giacomin et al., 2016). In recent years, nutritional intervention has become a new trend to maintain intestinal homeostasis. In this context, there has been an explosion of consumer interest in probiotics, and the market is increasing annually (Neef and Sanz, 2013). Probiotics are live microorganisms that, when administered in adequate amounts, confer a health benefit on the host (Hill et al., 2014). The benefits of probiotics include rebalancing the distribution of intestinal microbiota (Neef and Sanz, 2013), management of metabolic syndromes (Musso et al., 2010), and prevention of gastrointestinal diseases (Prakash et al., 2011; Whelan and Quigley, 2013). Studies have also shown that probiotics, such as *Lactobacillus, Bifidobacteriu*m, *Bacillus*, and *Enterococcus*, significantly inhibited pathogen infection *in vitro* and *in vivo* (Lebeer et al., 2008; Candela et al., 2008; Zhou et al., 2015; Safari et al., 2016). One possible mechanism of action is regulation of the immune response. Recent studies found that probiotics altered the inflammatory response by stimulating cytokine production (Ranadheera et al., 2014; Djaldetti and Bessler, 2017). However, further study of probiotics-mediated molecular mechanisms is still needed.

Autophagy is a highly conserved process in which cytoplasmic targets are sequestered in double membraned autophagosomes and subsequently delivered to lysosomes for degradation (Mizushima, 2011). Acting as an innate defense pathway in response to a variety of stimuli, autophagy is crucial for cytoplasmic recycling, fundamental homeostatis and cell survival (Nakagawa et al., 2004; Levine et al., 2011). Autophagy is also an essential component of the immune defense against bacterial pathogens such as *Mycobacterium tuberculosis, Salmonella enterica*, and *Escherichia coli* (Kirkegaard et al., 2004; Chargui et al., 2012; Bento et al., 2015). Thus, triggering autophagy in an appropriate manner is essential for cell survival during pathogens infection (Wang et al., 2013; Rekha et al., 2015).

The induction of autophagy involves numerous proteins and multiple signaling pathways. More than 30 members of the autophagy-related genes (Atg) family, such as *Beclin1* (homolog of *Atg6*), and Atg complexes are needed for autophagosome formation (Yang and Klionsky, 2010). Microtubule-associated protein 1 light chain 3 (LC3, a mammalian homolog of yeast *Atg8*), a vital component of the elongation step, is regarded as the autophagy marker (Mizushima, 2011). p62 (also known as SQSTM1), a common autophagy substrate and a marker of autophagic flux, is selectively incorporated into autophagosomes and efficiently degraded by autophagy (Xu et al., 2015). Moreover, several signaling pathways, including AKT (alpha serine/threonine kinase)/mTOR (mammalian target of rapamycin), AMPK [Adenosine 5′-monophosphate (AMP)-activated protein kinase], and MAPK (mitogen-activated protein kinase), modulate autophagy at different autophagosome formative stages (Yang and Klionsky, 2010).

Recent studies have suggested that probiotics can mildly regulate macrophages to enhance the immune response to foreign invaders. Macrophage, an important effector during pathogen invasion (Naito, 2008), can utilize autophagy to defend against infection (Martinez et al., 2013). Pathogens that are passively taken up by macrophages and stored in the cytosol or phagosome are eventually degraded by the autophagy pathway. A previous study found that probiotic mixture VSL#3 can induce a mixed inflammatory phenotype in macrophages (Isidro et al., 2014); *Lactobacillus acidophilus* and *Bacillus clausii* are potent activators of innate immune responses in murine macrophage cell line RAW264.7 cells (Pradhan et al., 2016). The immunostimulatory activity of probiotics depends on the interaction between microorganisms-associated molecular patterns (MAMPs) and toll-like receptors (TLRs) (Lebeer et al., 2010). This interaction is also involved in triggering autophagy in macrophages. Thus, probiotics may mediate antibacterial activity in macrophages through mechanisms that activate autophagy. Despite the evidence, only a few studies have explored the regulation of autophagy by probiotics (Kim et al., 2010; Wu et al., 2013; Lin et al., 2014), and its role in the elimination of pathogens is still unknown.

In the present study, we examined the relationship between probiotics and autophagy and its role in the elimination of pathogens. We found that probiotic *Bacillus amyloliquefaciens* SC06 (Ba) induced autophagy in RAW264.7 cells by upregulating the expression of Beclin1 and *Atg5-Atg12-Atg16* complex. This mechanism played a key role in protecting macrophages against *E. coli* infection.

## Materials and Methods

### Reagents

Antibody LC3 was obtained from Sigma–Aldrich (Sigma, St. Louis, MO, USA). phospho-ERK1/2 and anti-ERK1 were from BD Biosciences (San Jose, CA, USA). Antibodies including SQSTM/p62, phospho-AKT, AKT, phospho-mTOR, mTOR, Beclin1, phospho-JNK, and phospho-p38 were obtained from Cell Signal Technologies (Danvers, Massachusetts, USA). SAPK/JNK, p38, β-actin, HRP-conjugated anti-mouse IgG, and HRP-conjugate anti-rabbit IgG were from Beyotime (Shanghai, China). Alexa Fluor 488-conjugated secondary antibody to rabbit IgG was purchased from Life Technologies (Gaithersburg, MD, USA). Autophagy inhibitors chloroquine, 3-MA, and the activator rapamycin were purchased from Sigma–Aldrich (Sigma, St. Louis, MO, USA).

### Cell Culture and Bacteria Preparation

Murine macrophage cell line RAW264.7 were purchased from American Type Culture Collection (ATCC, Rockville, MD) and maintained in Dulbecco’s Modified Eagle Media (DMEM, Hyclone), supplemented with 10% fetal calf serum (FBS, Australian origin, Gibco), and 1% antibiotics (100 U/ml of penicillin G and 100 mg/ml of streptomycin) in a humidified atmosphere at 37°C. The probiotic Ba, was isolated from soil and preserved at China Center for Type Culture Collection (CCTCC, No: M2012280). Ba was grown in Luria-Bcrtani (LB) medium overnight at 37°C, harvested by centrifugation (5000 rpm, 10 min), washed 3 times and suspended in PBS at different optical densities at 600 nm (0.33 OD = 1 × 10^8^ cfu/ml). Then, bacteria were heated at 100°C for 30 min (Ji et al., 2013). The heat-killed bacteria precipitation was collected after centrifugation, and resuspended in DMEM for cell treatments. The *Escherichia coli* (*E. coli*) strain (C83549 (O149:k91, K88ac)) was obtained from China Institute of Veterinary Drug Control. *E. coli* expressing RFP (RFP-*E. coli*) was constructed in our lab. Both of them were grown in LB medium overnight at 37°C, and then incubated in fresh medium (1:100) for another 3 h for all experiments.

### Cell Cytotoxicity Assay

Cell viability was measured using cell counting kit 8 (CCK-8, Beyotime, China) (Ma et al., 2010). Briefly, monolayers of RAW264.7 cells were cultured in 96-well plates overnight and then pretreated with Ba at different concentrations (ranging from 10^6^ to 10^9^cfu/ml) for 12 h. Followed by removal of the cultured medium, 10 μL CCK-8 assay solution was added to every cell well and further incubated for 1 h. Subsequently, the OD value was measured using SpectraMax M5 at OD_450_ and the percentage of living cells was calculated as previously described (Mosmann, 1983). Lactate dehydrogenase (LDH) release from damaged cells was quantified using CytoTox96 kit (Roche Diagnostics, Mannheim, Germany) after cells co-cultured with 10^7^ or 10^8^ cfu/ml Ba for 12 h.

### Western blotting

Cells were lysed in RIPA buffer (Beyotime) on ice for 30 min. An equal amount of proteins (20 μg) from each sample were loaded on 8, 12, or 15% SDS-polyacrylamide gels and transferred to polyvinylidene difluoride (PVDF) membranes (Roche). After blocking with no protein blocking solution (SangonBiotech) at room temperature, the membranes were incubated with primary antibody overnight at 4°C. Following incubated with secondary antibody for 1 h, the immunoreactive bands were visualized with an ECL detection system. Densitometric quantification of band intensities was determined using Image J software.

### Immunofluorescence Staining Analysis

RAW264.7 cells were seeded on coverslips (Nest) in 12-well plates for overnight culture, and then treated with 10^8^ cfu/ml Ba or 2 μM rapamycin for 6 h. For infection assay, pretreated cells were infected with RFP-*E. coli* for 1 h in antibiotic free DMEM. Then, cells were fixed with cold methanol for 5 min, blocked with 2.5% BSA for 2 h in room temperature, and incubated with anti-LC3 antibody overnight at 4°C. After incubated with Alexa Fluor 488-conjugated antibody for 1 h, nuclei were labeled with DAPI for 10 min. Samples were mounted by confocal microscopy using the Olympus Laser Scanning Microscope (Olympus BX61W1-FV1000, Tokyo, Japan).

### Real-time PCR for Expression Analysis

Total RNA was isolated from treated RAW264.7 cells with RNAiso plus (Takara). cDNA was synthesized with PrimeScript II 1st Strand cDNA Synthesis Kit (Takara) according to the manufacturer’s instructions. Real-time PCR was performed using SYBR PremixExTaqII (Takara) and StepOnePlus real-time PCR system (Applied Biosystems). All samples were runned in duplicate. The gene expression levels were normalized to *β-actin* using the comparative Ct method (Schmittgen et al., 2000). The primers were as follows:

*Atg*5: forward, AGAAGATGTTAGTGAGATATGG
reverse, ATGGACAGTGTAGAAGGT;
*Atg*7: forward, AGCCCACAGATGGAGTAGCAGTTT
reverse, TCCCATGCCTCCTTTCTGGTTCTT
(Neal et al., 2013);
*Atg*12: forward, CCAAGGACTCATTGACTTC
reverse, GCAAAGGACTGATTCACATA;
*Atg*16: forward, TGTCTTCAGCCCTGATGGCAGTTA
reverse, AGCACAGCTTTGCATCCTTTGTCC.

### Antibacterial Assay

RAW264.7 cells were pretreated with Ba for 6 h, and infected with *E. coli* at a multiplicity of infection (MOI) of 30 for 1 h in antibiotic free medium. The antibacterial activity was assessed as previously described (Boudeau et al., 1999; Mao et al., 2015). In detail, cells were washed three times with PBS post infection, and cultured in medium containing gentamicin (100 μg/ml) for 1 h to eliminate extracellular bacteria. For phagocytosis analysis, cells were then lysed immediately in PBS with 1% Triton X-100. For bactericidal analysis, cells were lysed after a further incubation for 7 h or 19 h in medium containing 10 μg/ml gentamicin. The number of bacteria released from cells was detected by plating serial dilutions of the cell lysates on LB agar plates. Meanwhile, a portion of lysates was used to measure the concentration of cell protein. Bactericidal activity was analyzed by the remaining *E. coli* at each time point/cell protein concentration. All infections were performed in duplicate, and each experiment was repeated three times.

### Statistical Analysis

Data were expressed as means ± standard deviation. Differences were determined by two-tailed student’s *t*-test and one-way ANOVA using SPSS 20.0 statistical software (SPSS Inc., Chicago, IL, USA). *P*-values of <0.05 were considered statistically significant. All statistical analyses were performed using GraphPad Prism 5.0 software.

## Results

### Cytotoxicity Analysis of Ba on RAW264.7 Cells

**FIGURE 1:**
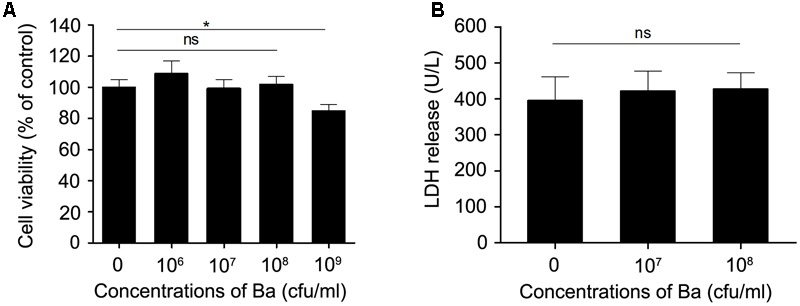
**Cytotoxity assay of *Bacillus amyloliquefaciens* SC06 (Ba) in RAW264.7 cells. (A)** RAW264.7 cells were exposed to Ba at different concentrations (10^6^, 10^7^, 10^8^, 10^9^ cfu/ml) for 12 h. Cell viability was determined by CCK-8 assay. **(B)** Cell damage was determined by measuring the release of LDH. RAW264.7 cells were incubated with Ba (10^8^ and 10^9^ cfu/ml) for 12 h, and LDH release in the supernatant was quantified using the CytoTox96 kit. Data are representative of three individual experiments. ^∗^*p* < 0.05 (*t*-test). ns indicates no significance (*p* > 0.05).

To evaluate the cytotoxicity of probiotics on macrophages, murine macrophage cell line RAW264.7 cells were treated with probiotic Ba. Cell viability was determined using the CCK-8 assay. No obvious decrease of viability was observed when cells were incubated with Ba at a range of concentrations (from 10^6^ to 10^8^ cfu/ml) (**Figure 1A**). Therefore, 10^7^ (97.5 ± 2.67 % viable cells) and 10^8^ cfu/ml (99.9 ± 2.47% viable cells) were used in subsequent experiments. To evaluate the effect of Ba on cell damage, we measured LDH activity in the cell culture supernatant. There was no significant difference in LDH activity following 12 h of treatment with Ba when compared to untreated cells (*p* > 0.05) (**Figure 1B**).

### Ba Stimulates Autophagy in RAW264.7 Cells

**FIGURE 2:**
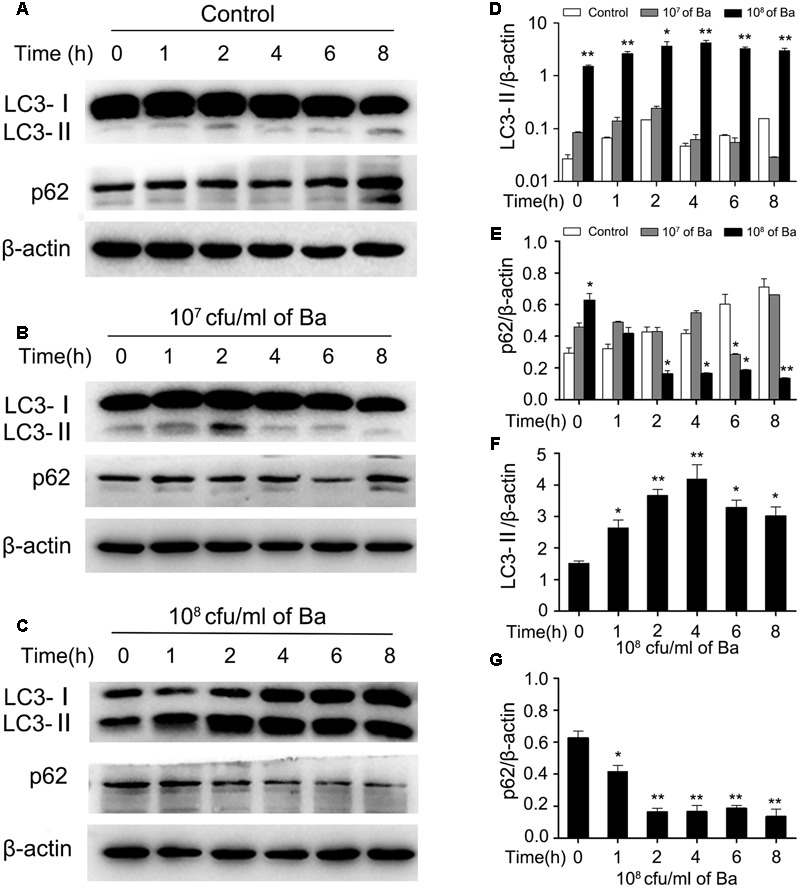
***Bacillus amyloliquefaciens* SC06 (Ba) increases LC3-II and degrades p62 in a dose- and time- dependent manner. (A)** Control: RAW264.7 cells were incubated from 0 to 8 h without any treatment, and were harvested and analyzed by western blotting using anti-LC3, anti-p62, or anti-β-actin antibody. **(B)** 10^7^ cfu/ml of Ba: Cells were treated with Ba at the concentration of 10^7^cfu/ml from 0 to 8 h. **(C)** 10^8^ cfu/ml of Ba: Cells were treated with Ba at the concentration of 10^8^cfu/ml from 0 to 8 h. **(D,E)** The ratio of LC3-II or P62 to β-actin were analyzed by Image J. Significance was determined by comparing to control cells. **(F,G)** The ratio of LC3-II or p62 to β-actin in 10^8^ cfu/ml of Ba group were analyzed by Image J. Significance was determined by comparing to the value at 0 h (*t*-test, ^∗^*p* < 0.05, ^∗∗^*p* < 0.01). Data are representative of three individual experiments with similar results.

**FIGURE 3:**
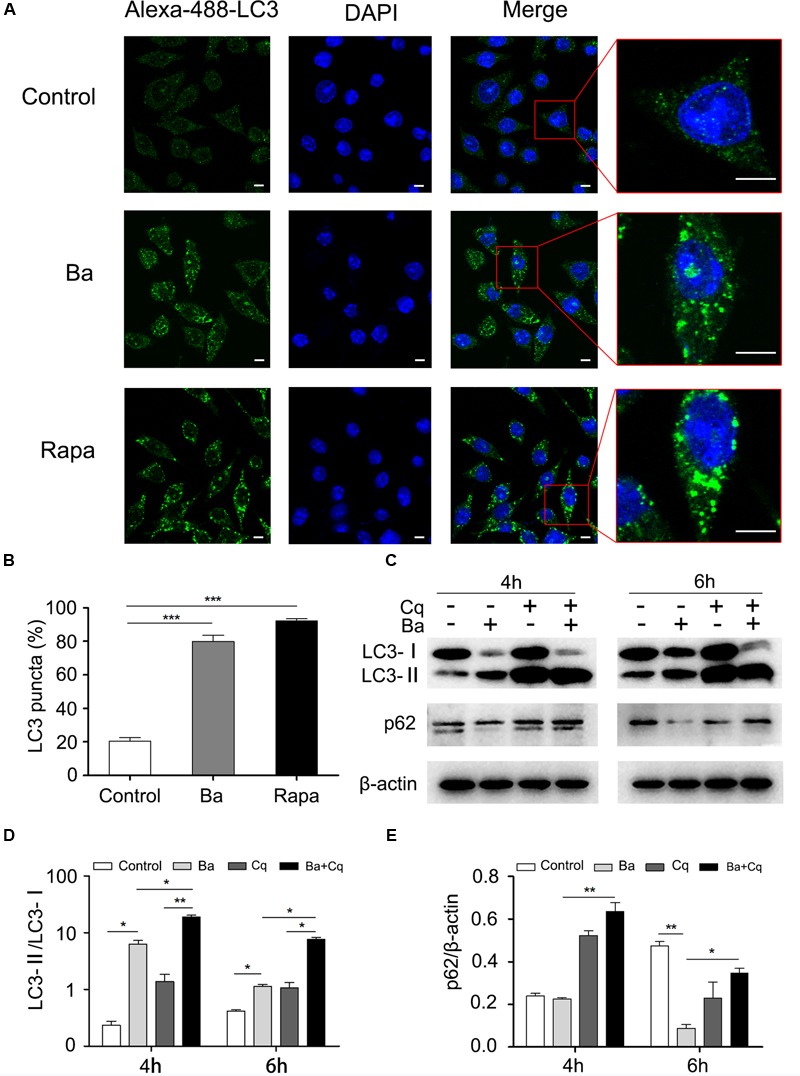
***Bacillus amyloliquefaciens* SC06 (Ba) treatment induces autophagy in RAW264.7 cells (A)** RAW264.7 cells were treated as follows. Control: Untreated cells. Ba: Cells were incubated with 10^8^ cfu/ml Ba for 6 h. Rapa: Positive control. Cells were treated with rapamycin (2 μM, 6 h). All cells were then stained using immunofluorescence technique and observed under a confocal microscopy. The scale bar represents 5 μm **(B)** Statistical analyses of the number of positive cells with > 3 green puncta. Values are from 100 cells per sample (*t*-test, ^∗∗∗^*p* < 0.001). Data are representative of three individual experiments with similar results. **(C)** Cells were pretreated with Chloroquine (Cq, 50 μM, 3 h) and then incubated with 10^8^ cfu/ml of Ba for 6 h. The levels of LC3 and p62 were identified by western blotting. **(D,E)** Analyses of LC3-II/LC3-I or p62/β-actin using Image J (one-way ANOVA; Tukey test, ^∗^*p* < 0.05, ^∗∗^*p* < 0.01). Data are representative of three individual experiments with similar results.

To determine whether Ba induces autophagy, RAW264.7 cells were incubated with Ba for different lengths of time (0, 1, 2, 4, 6, 8 h). Protein expression of Microtubule-associated protein 1 light chain 3 (LC3), an autophagy marker, was examined by western blotting (Klionsky et al., 2012). No significant changes in LC3-II expression were observed between untreated cells and cells treated with a low concentration (10^7^ cfu/ml) of Ba for 8 h (**Figures 2A,B,D**). However, LC3-II was significantly higher in cells treated with a high dose (10^8^ cfu/ml) of Ba compared to untreated cells (*p* < 0.01) (**Figures 2A,C,D**). Treatment with 10^8^ cfu/ml Ba upregulated intracellular LC3-II at 2 h (*p* < 0.05), peaked at 4 h (*p* < 0.01), and maintained high levels persistently up to 8 h (**Figures 2C,F**). In addition to LC3-II, we examined the autophagic flux by detecting degradation of p62, a common autophagy substrate. p62 expression significantly decreased from 2 h to 8 h (*p* < 0.05) in 10^8^ cfu/ml Ba-treated cells, but not in cells treated with 10^7^ cfu/ml Ba and untreated cells (**Figures 2E,G**). Autophagy induction was also confirmed with confocal laser scanning analysis of LC3 cells. As shown in **Figures 3A,B**, cells treated with 10^8^ cfu/ ml Ba or 2 μM autophagy activator rapamycin for 6 h significantly increased LC3 puncta (*p* < 0.001).

To further examine the effect of Ba on autophagy in macrophages, cells were treated with autophagy inhibitor chloroquine (Cq) prior to Ba treatment. Chloroquine, an agent that impairs lysosomal acidification, can block both the degradation of LC3-II and p62, leading to their accumulation (Tanida et al., 2005). Cells were pretreated with 50 μM Cq for 3 h, and then incubated with 10^8^ cfu/ml Ba for another 4 or 6 h. Treatment with Ba alone enhanced the conversion of LC3-I to LC3-II and the degradation of p62. However, pretreatment with Cq resulted in an increase in the ratio of LC3-II/LC3-I and inhibited p62 degradation (**Figures 3C–E**), confirming the activation of autophagy. Taken together, these findings suggest that Ba stimulates autophagy in macrophages.

### Ba Enhances the Elimination of *E. coli* in RAW264.7 Cells via Autophagic Pathway

**FIGURE 4:**
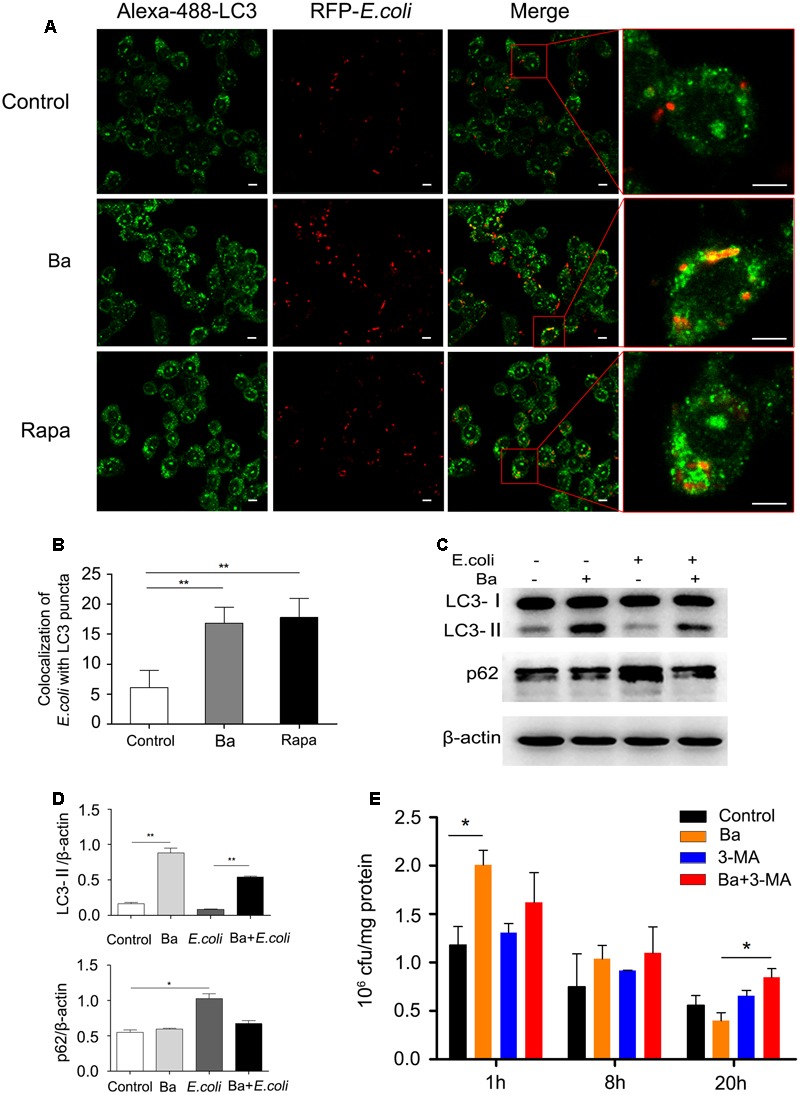
***Bacillus amyloliquefaciens* SC06-induced autophagy enhances the elimination of *E. coli* in RAW264.7 cells (A)** RAW264.7 cells were pretreated with 10^8^ cfu/ml of Ba or 2 μM rapamycin for 6 h, and then infected with RFP-*E. coli* for 1 h (MOI = 30). After immunofluorescence staining, the colocalization of *E. coli* with LC3 was observed by confocal microscope. The scale bar represents 5 μm. **(B)** Statistical analyses of the positive cells with >1 colocalization. Values are from 100 cells per sample (*t*-test, ^∗∗^*p* < 0.01). Data are representative of three individual experiments with similar results. **(C)** Cells were pretreated with 10^8^ cfu/ml Ba for 6 h, and then infected with *E. coli* (MOI = 30, 1 h). LC3 and p62 protein expression was determined using western blotting. **(D)** Analyses of the ratio of LC3-II or p62 to β-actin using Image J (one-way ANOVA; Tukey test, ^∗^*p* < 0.05, ^∗∗^*p* < 0.01). Data are representative of three individual experiments with similar results. **(E)** Cells were treated as follows. Control: Untreated cells. Ba: Cells were pretreated with Ba (10^8^ cfu/ml, 6 h). 3-MA: Cells treated with 3-MA (2 mM). Ba+3-MA: Cells pretreated with 3-MA (2mM, 3 h) and then incubated with Ba (10^8^ cfu/ml, 6 h). All the group were infected with *E. coli* (MOI = 30, 1 h). Following incubation with gentamicin for 1, 8, or 20 h, cells were lysed with 1% Triton X-100 in PBS, and the cfu was counted. Portions of the lysates were used to measure the concentration of cell protein. Remaining *E. coli* (cfu/mg) = Remaining *E. coli*/cell protein concentration. Values are from three independent experiments with similar results, one-way ANOVA, Tukey test, ^∗^*p* < 0.05.

To investigate whether Ba-induced autophagy is involved in antibacterial activity in RAW264.7 cells, we first analyzed the recruitment of LC3 to RFP-*E. coli.* Cells pretreated with 10^8^ cfu/ml Ba were infected with RFP-*E. coli* for 1 h. Compared to untreated cells, Ba-treated cells exhibited a markedly increased rate of *E. coli* colocalization with LC3 puncta (*p* < 0.01) (**Figures 4A,B**). Western blotting with total cell protein revealed that the ratio of LC3-II/β-actin was remarkably upregulated in Ba and Ba + *E. coli* cells, as compared with untreated and *E. coli* only treated cells (**Figures 4C,D**).

The phagocytosis and bactericidal activity in RAW264.7 cells were monitored by scoring bacterial colony forming units (cfu). As shown in **Figure 4E**, Ba significantly increased the uptake of *E. coli* (*t* = 1) (2.01 ± 0.15 × 10^6^ cfu/mg), compared with the control group (1.18 ± 0.19 × 10^6^ cfu/mg). Following 8 h incubation, the intracellular bacteria dropped but with no significance among all the groups. However, after 20 h, the number of *E. coli* in Ba-treated cells experienced a dramatic decrease (0.40 ± 0.08 × 10^6^ cfu/mg), compared to untreated cells (0.56 ± 0.10 × 10^6^ cfu/mg). Interestingly, when adding 3-MA to inhibit autophagy, antibacterial activity dramatically decreased, with 0.85 ± 0.09 × 10^6^ cfu/mg *E. coli* in Ba treated cells after 20 h. Taken together, the results suggest that Ba-induced autophagy enhances the elimination of *E. coli* in RAW264.7 cells.

### Ba Induces Autophagy by Upregulating the Expression of Beclin1 and *Atg5-Atg12-Atg16* Complex, But Not by AKT/mTOR Signaling Pathway

**FIGURE 5:**
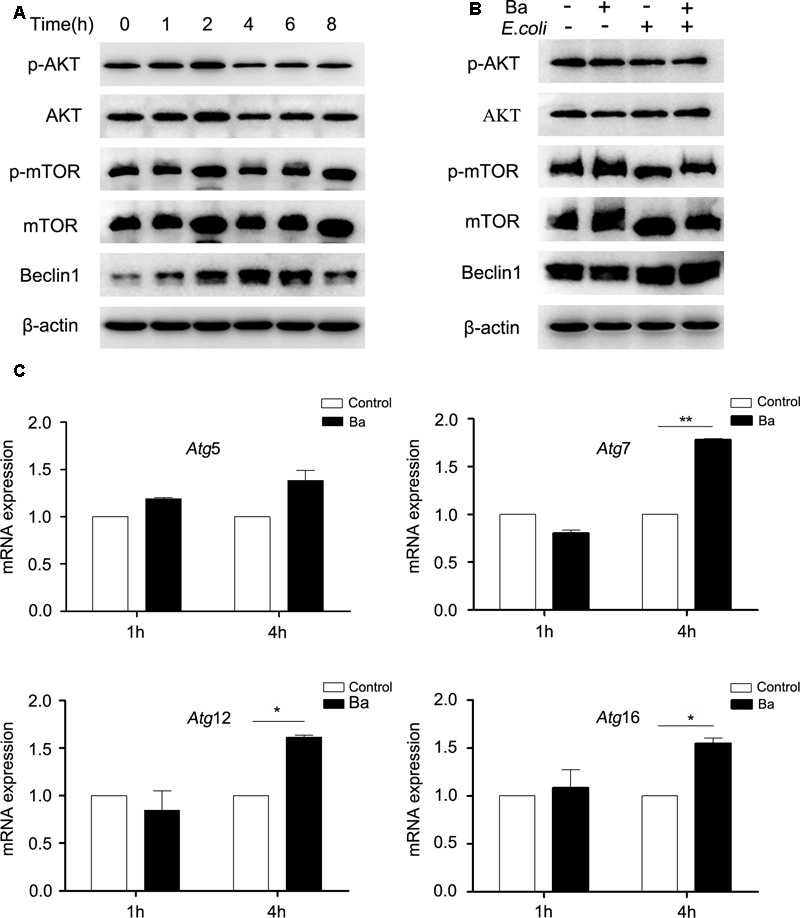
***Bacillus amyloliquefaciens* SC06 (Ba) upregulates the expression of Beclin1, but not the AKT/mTOR signaling pathway in RAW264.7 cells (A)** RAW264.7 cells were treated with 10^8^ cfu/ml Ba for 0 to 8 h. The cells were then harvested and analyzed by western blotting using anti-phospho-AKT, anti-AKT, anti-phospho-mTOR, anti-mTOR, Beclin1, or anti-β-actin antibody. **(B)** RAW264.7 cells were pretreated with Ba (10^8^ cfu/ml, 6 h), and then infected with *E. coli* (MO = 30, 1 h). Western analysis was the same as **(A). (C)** Cells were treated with Ba (10^8^ cfu/ml) for 1 or 4 h, and then the mRNA expressions of *Atg*5, *Atg*7, *Atg*12i, and *Atg*16 were detected by quantitative Real-Time PCR. Values are from three independent experiments with similar results, *t*-test, ^∗^ *p* < 0.05, ^∗∗^*p* < 0.01.

To elucidate the underlying mechanisms of Ba-induced autophagy, we examined the effect of Ba on autophagic signaling pathways (Beclin1 and AKT/mTOR). Cells were incubated with Ba alone for different lengths of time (0, 1, 2, 4, 6, 8 h), or pretreated with Ba for 6 h and subsequently challenged with *E. coli* for 1 h. Beclin1 is a core protein in autophagosome nucleation (McKnight and Yue, 2013). Results revealed that Beclin1 expression was upregulated in a time-dependent manner in response to Ba treatment alone (*p* < 0.05; **Figure 5A** and **Supplementary Figure S1C**). Consistently, pretreatment with Ba led to an increasing level of Beclin1 when cells were infected with *E. coli* (**Figure 5B** and **Supplementary Figure S1**). The inhibition of AKT/mTOR phosphorylation can activate autophagy (Mizushima, 2010). Western blotting analyses showed no significant changes of p-AKT/AKT and p-mTOR/mTOR after Ba treatment alone (*p* > 0.05, **Figure 5A** and **Supplementary Figures S1A,B**), and similar results could also be obtained during *E. coli* challenge (**Figure 5B** and **Supplementary Figures S1D,E**).

We also determined the mRNA expression of an autophagic complex *Atg5-Atg12-Atg16*, which is essential to LC3-II ligation to the autophagosome membrane (Klionsky et al., 2012). RAW264.7 cells were treated with Ba for 1 or 4 h. The mRNA expression levels of all the tested genes showed no differences after Ba treatment for 1 h (*p* > 0.05) (**Figure 5C**). However, after 4 h treatment, the mRNA expressions of *Atg*7 (*p* < 0.01), *Atg*12 and *Atg*16 (*p* < 0.05) increased markedly (**Figure 5C**).

All these observations suggest that Beclin1 and Atg5-Atg12-Atg16 complex may play important roles in the induction of autophagy in RAW264.7 cells by Ba treatment. However, the AKT/mTOR signaling pathway is not involved in Ba-induced autophagy.

### Ba Decreases the Phosphorylation of JNK Under Conditions of *E. coli* Infection in RAW264.7 Cells

**FIGURE 6:**
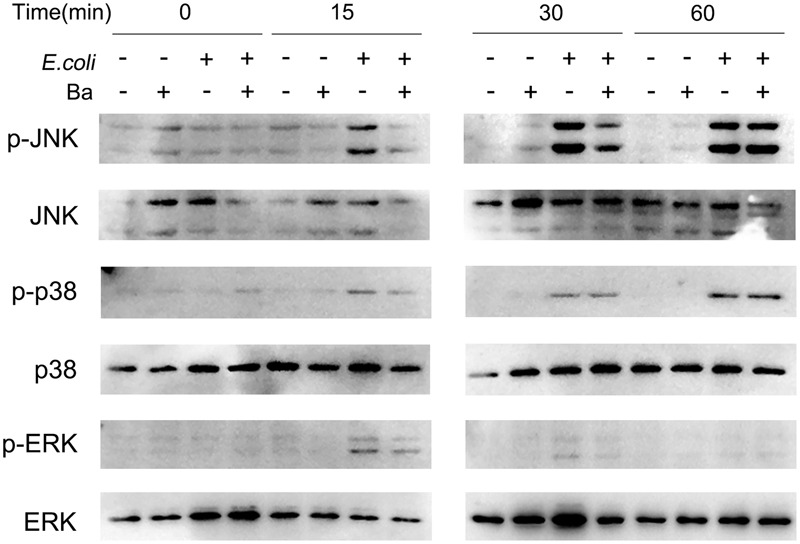
***Bacillus amyloliquefaciens* SC06 inhibits JNK phosphorylation in RAW264.7 cells.** RAW264.7 cells were pretreated with Ba (10^8^ cfu/ml, 6 h), and then infected with *E. coli* (MOI = 30) for 0, 15, 30, or 60 min. The expressions of phospho-JNK, JNK, phospho-p38, p38, phospho-ERK, and ERK were detected by western blotting.

As previous studies verified a critical role of MAPK signaling pathways in mediating microorganism–host interaction, we asked whether these pathways were activated in RAW264.7 cells during Ba treatment or *E. coli* infection. As shown in **Figure 6**, JNK phosphorylation remained at basal level during Ba treatment alone, but experienced a rapid increase when cells were challenged with *E. coli* after 15 min. Surprisingly, pretreated with Ba led to a dramatic decline in JNK phosphorylation. The accumulation of phospho-p38 in *E. coli*-infected cells started at 15 min and peaked at 60 min, but showed no significant difference when pretreated with Ba. Neither exposure to *E. coli* nor Ba significantly activated ERK phosphorylation, although a slight upward trend was observed at 15 and 30 min.

## Discussion

The relationship between probiotics and improved gut health has received considerable scientific interests for more than a century. Accumulating evidence supports the well-characterized immune modulation of probiotics in preventing intestinal diseases (Isaacs and Herfarth, 2008; Kleta et al., 2014; Lenoir-Wijnkoop et al., 2014). Macrophages, as important immune cells in intestine, when activated, can rapidly respond to pathogenic microorganisms by releasing inflammatory cytokines (Fernando et al., 2014). Our study indicates that probiotic can also enhance pathogen inhibition by triggering autophagy in macrophages, which provides valuable insights into the mechanism of probiotics in maintaining gut health.

Pathogens control is also one of the most important topics in food safety. In animal husbandry, the overuse of antibiotics can lead to severe drug resistance and increase food safety risks (Gilchrist et al., 2007). For these reasons, antibiotics have been forbidden in feed in Europe since 2006 (Chu et al., 2013). In order to produce safe and reliable animal products, probiotics such as *Lactobacillus, Bifidobacterium* and *Bacillus* are considered to be promising substitutes for antibiotics to prevent bacterial diseases (Zacarias et al., 2014; Yang et al., 2015; Guo et al., 2016). *Bacillus amyloliquefaciens*, belonging to *Bacillus* genus, is a species closely related to *Bacillus subtilis* (Xu Z. et al., 2013). With strong bactericidal activity to suppress numerous pathogens (fungi and bacteria) (Xu Z. et al., 2013; Wu et al., 2014; Torres et al., 2017), *B. amyloliquefaciens* strains, are not only widely used as plant growth-promoting rhizobacteria (PGPR) and biocontrol agents in agriculture (Soylu et al., 2005; Chowdhury et al., 2015), but also have been attracted to be potential biopreservative in food industry (Kaewklom et al., 2013; Wang et al., 2014). In recent years, an increasing number of reports further their beneficial effects on the growth performance and infectious disease resistance of animals when being used as probiotics (Gracia et al., 2003; Das et al., 2013; Thy et al., 2017). Our previous trials found that supplement with Ba inhibited *E. coli*-induced pro-inflammatory responses and alleviated diarrhea in weaned pigs (Ji et al., 2013). Similarly, *B. amyloliquefaciens* protected against *Clostridium difficile*-associated disease in a mouse model (Geeraerts et al., 2015). Previous studies highlighted that *B. amyloliquefaciens* strains exerted antagonistic activity against pathogens by producing diverse bioactive metabolites including lipopeptides, fengycin, and iturin (Wong et al., 2008). Here we found that heat-killed Ba itself could trigger immune response and protect against pathogens, this deepen the mechanisms of their antimicrobial effects and further their use as probiotics.

Our study is the first to show that probiotic-mediated autophagy contributes to bacterial inhibition by macrophages. Using western blotting and confocal laser scanning analysis, we found that treatment with Ba induced autophagy-related processes including LC3-II accumulation, p62 degradation, and LC3 puncta aggregation in RAW264.7 cells. Furthermore, cells pretreated with Cq, an inhibitor of autophagic flux, facilitated the ratio of LC3-II/LC3-I and the accumulation of p62 during Ba treatment. Taken together, these findings demonstrate Ba can act as a stimulant of autophagic activity in RAW264.7 cells.

We determine that probiotic-induced autophagy inhibits *E. coli* growth in RAW264.7 cells. Pretreatment of infected cells with Ba remarkably elevated the LC3-II expression, phagocytosis, co-localization of RFP-*E. coli* within autophagosomes and *E. coli* elimination. Moreover, 3-MA blockade of autophagy dramatically impaired bactericidal activity. Thus, we confirm autophagy activated by Ba contributes to the inhibition of intracellular *E. coli.* Consistent with our findings, other investigators have reported that enhanced autophagy played a role in pathogen elimination. Wang et al. (2013) revealed LPS-induced autophagy was a cell-autonomous defense mechanism involved in the restriction of *E. coli* in peritoneal mesothelial cells. Another study demonstrated poly(I:C)-induced autophagy mediated the elimination of *mycobacteria* in macrophages (Xu Y. et al., 2013). Physiological induction of autophagy or its pharmacological stimulation by rapamycin could suppress intracellular survival of *mycobacteria* in infected macrophages (Gutierrez et al., 2004).

**FIGURE 7:**
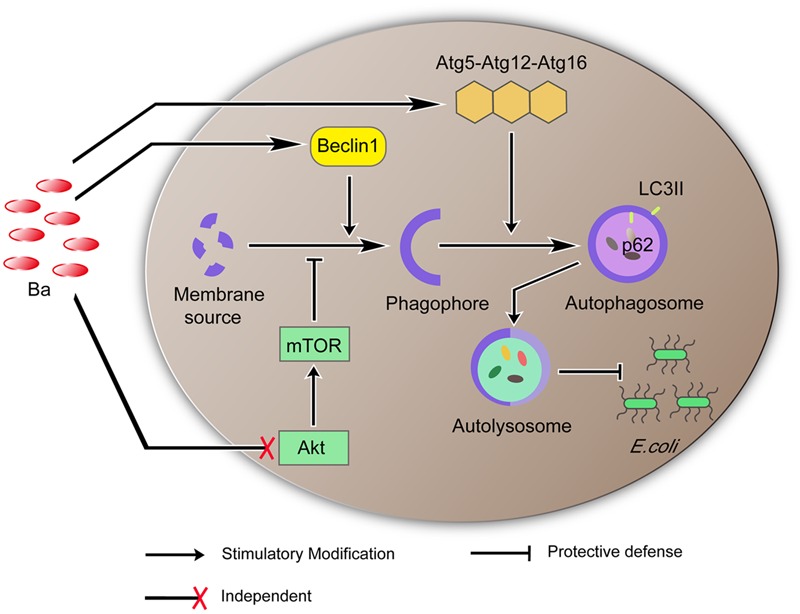
**Proposed model of Ba-induced autophagy and its function in eliminating *E. coli*.** Ba upregulates the expression of Beclin1. Beclin1 promotes formation of the phagophore. The phagophore incorporates the *Atg*5*-Atg*12*-Atg*16 complex into its membrane to generate an autophagosome which consumes and lyses invading pathogens. AKT/MTOR is independent in this process.

We also determine that Ba alters expression of several signaling pathways and proteins that regulate autophagy. Beclin 1, as a core component of class III phosphatidylinositol 3-kinase (PI3K-III) complex, enables recruitment of a number of autophagy proteins involved in the nucleation of autophagosome (McKnight and Yue, 2013). The Atg5-Atg12-Atg16 complex is a ubiquitin-like complex that is required in the final step of autophagosome formation, elongation of isolation membrane and/or completion of enclosure (McKnight and Yue, 2013). We found that Ba significantly upregulated Beclin1 expression in a time-dependent manner in RAW264.7 cells. Beclin1 expression also experienced an uptrend when pretreated with Ba during *E. coli* infection. The mRNA expression levels of *Atg7, Atg12, Atg16* increased significantly after Ba treatment. Additionally, one of the most conserved autophagy pathways is dependent on the metabolic checkpoint kinase mTOR, which can be initiated by AKT (Laplante and Sabatini, 2012). During nutrient starvation or other stress, the activity of AKT/mTOR is inhibited, resulting in translocation of ULK complex (ULK1/2, Atg13, FIP200, and Atg101) which activates autophagy (Mizushima, 2010). Surprisingly, our study showed that phosphorylations of mTOR and AKT were not decreased by Ba treatment alone or subsequent *E. coli* infection. mTOR is assembled and functional only when cellular nutrients or cofactors are not limited (Laplante and Sabatini, 2012). Unlike some pathogens, such as *Salmonella* or *Listeria*, may trigger a rapid inhibition of mTOR signaling through competition for nutrients (Tattoli et al., 2012, 2013). Ba delivers a mild stimulus to cells and no nutrients competition, thus it might explain no reduction of phosphorylation levels of AKT and mTOR. Taken together, the signaling pathways involved in the activation of autophagy by Ba were not dependent in AKT/mTOR, but possibly via regulating expressions of Beclin1 and *Atg5-Atg12-Atg16* complex (**Figure 7**).

Mitogen-activated protein kinase signaling pathway is one of the most important regulators of physiological cell processes including inflammation, stress, cell growth, differentiation and death. JNK is considered to be activated by a number of stressors which can induce apoptosis or growth inhibition (Bogoyevitch and Kobe, 2006). Our study showed that JNK phosphorylation in RAW264.7 cells increased after 15min, and lasted up to 60 min when infected with *E. coli*. Similarly, studies showed that infected with *S. Flexneri* for 20 min increased JNK activation in HeLa cells (Girardin et al., 2001) and *E. caratavora* induced JNK phosphorylation after 30 min in *Drosophila* larvae (Jones et al., 2008). During bacterial infection, the rapid activation of JNK initiates nuclear factor activator protein-1 (AP-1) to regulate pro-inflammatory cytokines expression, which could trigger excessive inflammation (Weston and Davis, 2007; Zhang et al., 2013). Interestingly, we observed that pretreatment with Ba for 6 h inhibited the activation of JNK. Similar results were found in other probiotics. For example, *Lactobacillus* attenuated the expression of pro-inflammatory cytokines caused by *E. coli* challenge by downregulating JNK activation in Caco-2 cells (Yu et al., 2015); Increased JNK activity in obese mice was abolished during probiotic administration (Toral et al., 2014). According to previous and our results, we can deduce that, the suppression of JNK activity by Ba has a protective effect during *E. coli* infection and Ba might play an anti-inflammatory role in RAW264.7 cells. Furthermore, we investigate whether the inhibition of JNK is associated with autophagy. Autophagy is involved in both cell death and cell survival depending on the cell type and strength of specific stimuli (Janku et al., 2011). A previous study demonstrated that JNK activation mainly contributed to autophagic cell death, which eventually caused cell apoptosis (Bowrsello et al., 2003). Therefore, we speculate that probiotic Ba, as a mild activator, triggers cell protective autophagy and enhances the immune function of RAW264.7 cells during *E. coli* challenge by suppressing JNK phosphorylation and inhibiting *E. coli*-induced pro-inflammatory responses.

In summary, the present study reveals that heat-killed probiotic Ba activates autophagy via upregulating the expression of Beclin1 and *Atg5-Atg12-Atg16* complex and that the induced-autophagy promotes the elimination of *E. coli* in RAW264.7 cells. Moreover, Ba reduces the levels of JNK phosphorylation triggered by *E. coli*, indicating an anti-inflammatory role of Ba. To our knowledge, this is the first report to uncover probiotic-mediate autophagy enhancement of the antibacterial activity of macrophages. These findings deepen our understanding of the immune protective capabilities of probiotics and may aid in the application of probiotics in the food industry to improve human or animal’s health. However, whether these protected mechanism function *in vivo* warrants further investigation.

## Author Contributions

WL, XX, and QS conceived and designed the experiments; YpW, YW, AF, YyW, and YbW performed the experiments; YW analyzed the data; YpW and BW made the figures; YpW, HZ, and YW wrote the paper.

## Conflict of Interest Statement

The authors declare that the research was conducted in the absence of any commercial or financial relationships that could be construed as a potential conflict of interest.

## Supplementary Material

FIGURE S1 **(A–F)** Densitometric analyses of p-AKT/AKT, p-mTOR/mTOR, and Beclin-1/β-actin in **Figures 5A,B**. Values are from three independent experiments with similar results, one-way ANOVA, Tukey test, ^∗^*p* < 0.05.
