# Supplementary figures and images for: Probiotic Bacillus amyloliquefaciens SC06 Induces Autophagy to Protect against Pathogens in Macrophages
Source: Front Microbiol. 2017 Mar 22;8:469. doi: 10.3389/fmicb.2017.00469 (PMC5360707; doi:10.3389/fmicb.2017.00469)

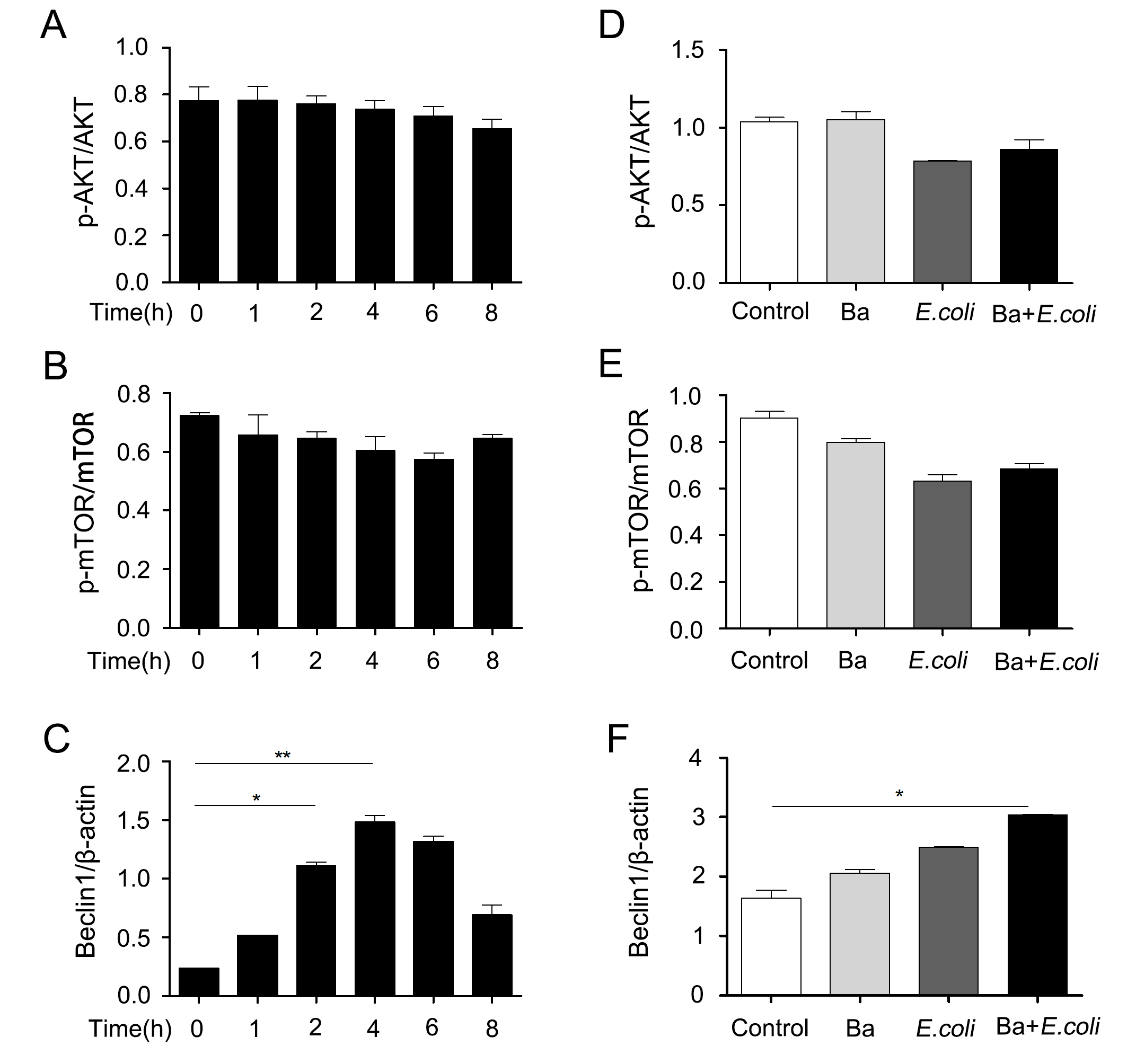

Supplement: FIGURE S1 — (A–F) Densitometric analyses of p-AKT/AKT, p-mTOR/mTOR, and Beclin-1/β-actin in Figures 5A,B. Values are from three independent experiments with similar results, one-way ANOVA, Tukey test, ∗p < 0.05. [file Image_1.TIF]
